# What’s in a cue?: Using natural language processing to quantify content characteristics of episodic future thinking in the context of overweight and obesity

**DOI:** 10.1080/21642850.2025.2510417

**Published:** 2025-06-02

**Authors:** Haylee Downey, Shuangshuang Xu, Sareh Ahmadi, Aditya Shah, Jeremiah M. Brown, Warren K. Bickel, Leonard H. Epstein, Allison N. Tegge, Edward A. Fox, Jeffrey S. Stein

**Affiliations:** a Fralin Biomedical Research Institute at VTC, Roanoke, VA, USA; b Graduate Program in Translational Biology, Medicine, and Health, Virginia Tech, Blacksburg, VA, USA; c Department of Computer Science, Blacksburg, VA, USA; d Department of Pediatrics, University at Buffalo Jacobs School of Medicine and Biomedical Sciences, Buffalo, NY, USA; e Department of Human Nutrition, Foods, and Exercise, Virginia Tech, Blacksburg, VA, USA

**Keywords:** Delay discounting, episodic future thinking, natural language processing, intervention effectiveness, type 2 diabetes, obesity

## Abstract

Episodic future thinking (EFT), an intervention in which participants vividly imagine their future, has been explored as a cognitive intervention to reduce delay discounting and decrease engagement in harmful health behaviors. In these studies, participants generate text descriptions of personally meaningful future events. The content of these text descriptions, or cues, is heterogeneous and can vary along several dimensions (e.g. references to health, celebrations, family; vividness; emotional valence). However, little work has quantified this heterogeneity or potential importance for EFT’s efficacy. To better understand the potential impact of EFT content in the context of health behavior change (e.g. diet) among people with or at risk for obesity and related conditions, we used data from 19 prior EFT studies, including 1705 participants (mean body mass index = 33.1) who generated 9714 cues. We used natural language processing to classify EFT content and examined whether EFT content moderated effects on delay discounting. Cues most commonly involved recreation, food, and spending time with family, and least commonly involved references to health and self-improvement. Cues were generally classified as highly vivid, episodic, and positively valent (consistent with the intervention’s design). In multivariate regression with model selection, EFT content did not significantly moderate the effect of the episodic thinking intervention. Thus, we find no evidence that any of the content characteristics we examined were important moderators of the efficacy of EFT in reducing delay discounting. This suggests that EFT’s efficacy is robust against variability in these characteristics. However, note that in all studies, EFT methods were designed to generate high levels of vividness, episodicity, and emotional valence, potentially resulting in a ceiling effect in these content areas. Moreover, EFT content was not experimentally manipulated, limiting causal inference. Future studies should experimentally examine these and other content characteristics and evaluate their possible role in EFT’s efficacy.

Over 40% of adults in the United States have obesity (Stierman et al., 2021), putting them at risk for chronic diseases later in life, such as type 2 diabetes. Obesity has many causes, including the food environment, genetics, and diet (Masood & Moorthy, 2023; Swinburn et al., 2011; E. P. Williams et al., 2015). Targeting behavioral factors that influence excess energy consumption is one approach to prevent and treat diet-related chronic diseases. One behavioral factor implicated in diet-related behaviors and obesity is delay discounting, the tendency for delayed outcomes to be devalued (Odum et al., 2020). Greater devaluation of delayed outcomes has been associated with lower diet quality of foods purchased and consumed (Appelhans et al., 2019; Epstein et al., 2022), including lower vegetable consumption (Sukumar et al., 2022) and greater soft drink consumption (Kulbida et al., 2024). In addition, higher body mass index has been associated with greater delay discounting, albeit with substantial heterogeneity across studies (Amlung et al., 2016; Tang et al., 2019). Interventions that target delay discounting may help expand people’s temporal horizon and increase valuation of future outcomes, thereby increasing engagement in health behaviors that are only reinforced after long delays (Rung & Madden, 2018).

Episodic future thinking (EFT), or the simulation of prospective events (Rösch et al., 2022; Schacter et al., 2017), has been used as a behavioral intervention to reduce delay discounting and increase beneficial health behaviors (Brown & Stein, 2022). EFT interventions involve vividly imagining positive events in one’s future. Usually in EFT, participants are asked to write a short description of the event they are imagining, including details like who is there, what they are doing, and how they feel. These text descriptions, or *cues*, are then used to prompt EFT during the completion of behavioral tasks or in the natural environment. In populations who are overweight or with obesity, prediabetes, or type 2 diabetes, EFT has been shown to reduce delay discounting (for review see Colton et al., 2024). Studies have also examined the effect of EFT on other measures related to diet and eating behaviors, including the reinforcing value of unhealthy food (Athamneh et al., 2021; Bickel et al., 2020; Stein et al., 2021), foods purchased while grocery shopping (Hollis-Hansen et al., 2019; Hollis-Hansen et al., 2020; Hollis-Hansen et al., 2020), and craving (Athamneh et al., 2021). However, like many interventions, the impact of EFT on delay discounting and other outcomes varies across participants, leaving questions as to mechanisms that are associated with positive treatment effects, as well as individual differences in the outcomes.

One factor that could explain differences in EFT efficacy is what participants imagine in their events. The *content specificity hypothesis* poses that the effects of an intervention depend on the cue content and that cue content related to a target behavior or future payoff is more impactful than other cue content (Rösch et al., 2022). Some studies have experimentally manipulated EFT cue content to probe this hypothesis and similar ones (Athamneh et al., 2021; Epstein et al., 2021; Olsen et al., 2024; O’Donnell et al., 2017, 2019). For example, in a study comparing the effects of alcohol-related and non-alcohol-related EFT on delay discounting in a sample of undergraduates who drink, Chang and Ladd (2023) reported that non-alcohol-related cues reduced delay discounting relative to a control, but alcohol-related EFT cues did not. One study found that engaging in EFT with or without food content resulted in similar reductions in delay discounting, but EFT with food-related content resulted in larger reductions in eating-related outcomes (Dassen et al., 2016). However, it is unclear if the food content was related to a target behavior or the types of food content participants imagined.

Although we advocate for this experimental approach, at least three limitations are evident in this prior work. First, despite growing interest in the use of EFT to facilitate weight loss and diabetes prevention (Liu et al., 2023), very few studies have experimentally examined cue content in participants with overweight/obesity. The extent to which prior experimental findings may generalize across populations is unknown. Second, with some exceptions (e.g. Chang & Ladd, 2023), experimenters generally have not examined the extent to which participants’ text descriptions match the intended experimentally assigned content areas. As a result, the treatment fidelity of many experimental manipulations has not been evaluated. A technology is needed to quantify EFT content characteristics while minimizing bias from human observers. Third, and finally, many experiments would need to be conducted to cover all relevant cue content areas, which may prevent the discovery of important variables that moderate EFT’s efficacy on DD.

Using another approach, meta-regressions have investigated how cue characteristics moderate the impact of EFT on delay discounting and health relevant outcomes by coding study-level characteristics such as vividness and emotional valence (Colton et al., 2024; Rösch et al., 2022). These reviews suggest greater cue vividness is associated with a stronger EFT effect. However, these study-level analyses are limited because they are only able to consider coarse, study-level variability in content characteristics, which is likely less sensitive to moderating effects than analysis of participant-level content variability. Study-level analyses are further limited because they can only examine cue characteristics measured and reported in the original study, which may not include all characteristics of interest (e.g. the combined effects of subject matter and vividness).

A complementary approach that addresses these limitations is to use natural language processing (NLP) to quantify cue content characteristics of individual cues from prior data sets, followed by examination of each content area as a potential moderator of observed effects. NLP approaches have been used in similar contexts where participants generate large amounts of text, such as in scoring autobiographical interviews (van Genugten & Schacter, 2024). Although this observational approach may be vulnerable to confounding variables (e.g. active people discussing exercise more), it may accelerate identification of potentially important EFT content areas, which may be followed using experimental manipulations in confirmatory research. Ultimately, understanding whether individual cue characteristics moderate the behavioral effects of EFT may lead to more targeted and effective versions of the intervention in future research.

In the present study, we compiled cue, demographic, and delay discounting data from 19 studies our labs conducted on EFT, and used natural language processing to: (1) quantify the content of EFT cues, and (2) determine if any cue content characteristics moderate the effects of EFT on delay discounting. This data set comprised 1705 participants and 9714 cues. Due to growing interest in the use of EFT to change delay discounting in the context of clinical interventions for weight loss, we focused primarily on prior studies that recruited participants with overweight or obesity. Studies recruiting participants with substance use (e.g. Athamneh et al., 2022) were not included, but we did include some studies from the general population given the high rate of obesity and overweight in the US and relevance of health behaviors like diet and exercise to the vast majority of people.

## Method

Ethics Approval Statement: The Virginia Tech Institutional Review Board (IRB) designated this study (IRB# 22-832) as ‘not human subjects research’ and it was therefore exempt from IRB review.

### Data Sources and Compilation

All data sources were those from prior studies conducted by our research group (Virginia Tech and University at Buffalo). No other research teams were contacted for data and we did not search for potentially relevant datasets on open science platforms. Studies were with participants in the general population, with an emphasis toward samples with overweight, obesity, prediabetes, or type 2 diabetes. To minimize potential heterogeneity across participants and cue content, we did not include any study in which the sample was primarily people with substance use. Further requirements were that the study design included randomization to EFT or a control condition and that subject-level data was available for further analyses. For each study, when available, we obtained unprocessed data (i.e. from Qualtrics). We used inclusion criteria as described in published manuscripts by the original authors (see Supplementary Materials for list of studies). We extracted cues, demographics, and measures of delay discounting from each participant. Due to shared data collection instruments across labs for many studies, only minimal changes to levels of demographic variables were required to harmonize variables across studies. For delay discounting, when possible, we obtained indifference points rather than pre-calculated outcome measures. For each study, we calculated *k* values using Mazur’s (1987) equation, where D is the delay in days for all studies. For studies that used multiple amounts in delay discounting tasks, we used $100 if available. If multiple types of delay discounting tasks were used, we used the adjusting-amount titrating task rather than adjusting delay task since the adjusting-amount task provides more information.

Studies included in this dataset vary in their design, population, study type, sample size, EFT administration, and delay discounting measurement. Designs include EFT vs. control(s) and designs with EFT vs. control, plus another manipulation. For control conditions, most studies used episodic recent thinking (ERT) or standardized episodic thinking (SET). ERT is similar to EFT, except that participants are asked to consider events that occurred in the recent past. In SET, all participants have a similar past experience (playing video games in a laboratory setting). These control conditions are generally thought to be matched to EFT in length and valence, although limited work has systematically compared other aspects of these cues (e.g. content). Populations included people with overweight or obesity (*n* = 15; with or without prediabetes, type 2 diabetes, or other comorbidities), participants from the general population (*n* = 3), and with lower income (*n* = 1). Some studies were acute laboratory or online studies where participants were only exposed to EFT once. In some studies, participants were treatment seeking and engaged in EFT multiple times. Sample sizes for each study range from 7 to 262. For EFT administration, participants generated cues at various timeframes and the number of cues generated in each study also varied from 1 to 10 (see Supplementary Figure 1). In addition, in some studies how EFT was delivered (e.g. the number of cues) was experimentally manipulated. Delay discounting was measured using an adjusting-amount task in all but one study; all but three studies measured delay discounting of a hypothetical $100; and the longest delay in the delay discounting task ranged from 1 year to 25 years. Of note, some studies did not measure delay discounting, so we do not use these participant data in moderation analyses (*n* = 271). See Supplementary Table 4.

### Measures

#### Individual-level variables

For demographics, we extracted household income (continuous), education (ordinal), age (continuous), gender (nominal), ethnicity (binary), and race (nominal) variables. We treated responses of prefer not to say (or similar responses) as missing. We also obtained body mass index (BMI) for each participant. We restricted the realistic range of BMI values from 12-70, and treated values outside that range as missing (extreme values were likely due to data entry error). For BMI and demographics, we imputed missing values with the mean/mode value from the entire dataset. Nominal demographic variables were dummy-coded. For delay discounting, we use the natural log of *k*. Participants with missing delay discounting were excluded. The variable ‘Thinking Condition’ refers to if the participant experienced EFT or a control (i.e. Not EFT; e.g. no cue control, standardized episodic thinking, or recent episodic thinking).

#### Cue-level content characteristics

For each cue, we have predicted probabilities from NLP classifiers for the following binary concepts: Family, Friends, Romantic Partner, Solo, Health, Celebration, Recreation, Self-Improvement, Food; and the following trinary concepts: Emotional Valence, Vividness, Episodicity (Ahmadi & Fox, 2023; Shah, 2023). These classifiers are summarized in Table 1. Example cues for each content characteristic are provided in Supplementary Table 1. NLP classification accuracy metrics can be found in Supplementary Tables 2 and 3.

**Table 1.** Natural language processing classifier descriptions.

Concept	Meaning
**Cue-level, binary classifiers**	
Family	Family members mentioned
Friends	Friend mentioned
Romantic Partner	Romantic partner mentioned
Solo	Writer indicates they are alone
Health	Contains an obvious or specific reference to physical or mental health, including (but not limited to) improved or worse physical state or mental health, intentional changes in behaviors to improve health (e.g. eating vegetables with dinner), and health outcomes (e.g. diabetes status or weight)
Celebration	Contains an obvious, specific reference to a celebration or a celebratory event. Examples include but are not limited to graduations, birthdays, anniversaries, weddings, parties, and holidays such as Christmas and Thanksgiving.
Recreation	Contains obvious or specific references to engaging in an activity for leisure or fun while not working at one’s job. Examples include but are not limited to sports or physical activities like running or hiking, art, movies and television, or hobbies like gardening.
Self-Improvement	Contains an obvious or specific reference to ‘a better me’, including personal development, self-improvement, making positive changes in life, achievements, hard work, or determination. May contain references to the idea that things are looking up or getting better.
Food	Contains an obvious or specific reference to food, eating, cooking, or a meal. Eating or food is a major and essential component of the text.
**Cue-level, trinary classifiers**	
Emotional Valence	The extent to which the cue contains references to positive emotions and behaviors including laughing, smiling, and happiness rather than negative emotions or behaviors including sadness, crying, or anger.
Vividness	The extent to which the text contains sufficient and specific details so that the event described is readily and easily imaginable. The amount of context given regarding the event.
Episodicity	The extent to which the writer describes personal experiences, events, and actions rather than general facts or ideas. The extent to which the writer describes the events as if they are currently experiencing them ‘in the moment’. The extent to which the writer provides details about their emotions and/or what they hear, see, or feel.
**Cue set-level, continuous classifiers**	
Similarity (Classifier-Based)	The extent to which cues within a set discuss similar binary concepts.
Similarity (Semantic-Based)	The extent to which cues within a set are semantically similar.

Emotional Valence, Vividness, and Episodicity were examined because these characteristics are theorized to be key components of EFT (Rösch et al., 2022) and, accordingly, participants are instructed to write cues that are positive, vivid, and contain episodic details. Concepts about people were included because participants in EFT and ERT conditions are instructed to describe who they are with in the event described in the cue. Recreation, Celebration, Health, and Self-Improvement were identified as prevalent themes using other NLP approaches (e.g. text-based clustering, Semantic Search) and thus were included in the interest of describing cue content. Health, Self-Improvement, and Food were examined because previous studies have hypothesized that these factors may modify the impact of EFT and have used experimental methods to assess the impact of these, or similar, content areas (Athamneh et al., 2021; Dassen et al., 2016; O’Donnell et al., 2017).

Each binary classifier model assigns each cue a predicted probability that a cue contains or does not contain the content. Values closer to 0 represents low probability that the cue contains that characteristic and closer to 1 represents high probability that the cue contains that characteristic. Rather than use a cutoff and discretely classify each cue into a group (e.g. has food or does not have food), we use the predicted probability in moderation analyses to incorporate any ambiguity about which group the cue would be assigned to. For trinary concepts, cues were assigned one of three possible categories. For Emotional Valence, levels were Negative, Neutral, and Positive. For Vividness and Episodicity, levels were Not, Moderately, and Highly. To perform calculations (e.g. means) with these trinary variables, each label was assigned a value of 0, 0.5, and 1, respectively. Importantly, all classifiers were applied independently. Thus, each cue has a predicted probability value for each classifier (i.e. a single cue can have a high value for more than one concept).

#### Cue set-level content characteristics

Similarity scores were calculated in an attempt to operationalize narrativity across a set of cues, which has been previously examined experimentally (Epstein et al., 2021). However, note that similarity is likely a more accurate interpretation of these scores than narrativity per se. To measure if participants wrote about similar content across their cues, we calculated 2 indices of similarity for cue sets. For Similarity (Classifier-Based), we used predictions from NLP classifiers to generate an 11-element vector representing the classifier's probability prediction for 11 binary categories (9 are reported on in here; see Supplementary Materials for details). For each set of related cues, the vector elements are averaged across all cues to produce a mean vector V. Cosine similarity is computed between each cue’s vector and V, with the final output being the average of these similarities. The possible range of Similarity (Classifier-Based) is 0 to 1, with higher values indicating more similarity. For Similarity (Semantic-Based), we considered the language used by participants across cues. We construct a 768-element vector for each cue using the Sentence-BERT embedding model (Reimers & Gurevych, 2019). For cues within a set, the vectors are averaged to create a mean vector, V. Cosine similarity is then calculated between each cue's vector and V, with the final output being the average similarity score. The Similarity (Semantic-Based) ranges from 0 to 1, with higher values indicating greater semantic similarity among the cues in the set.

### Data analysis

We registered a statistical analysis plan on the Open Science Framework: https://osf.io/n46w3/?view_only = af3dde2ae7e744cb95ccfc9116ea8bf5

#### Descriptive statistics

All analyses were conducted using R and R Studio. For demographics, we calculated the mean (SD) for continuous variables or *N* (%) for categorical variables using the R package `tableone` (Yoshida & Bartel, 2022). For cue content characteristics, each participant has a different number of cues at different timeframes, making it difficult to use cue-level variables as predictors in analyses. To facilitate analysis, we calculated the mean value for each cue characteristic across all of a participants’ cues to arrive at a value for each participant’s cue set. We elected to take the mean across cue sets so that participants have the same number of cue content predictors and not hundreds of predictors with a substantial amount of missing data. To address potential disadvantages of using the mean, we also calculated the minimum and maximum value for each content characteristic across participants’ cue sets to use in analyses.

To describe cue characteristics (mean of all participants’ cue set values), we calculated means and SDs for participants with and without delay discounting measurements using the R package `tableone`. We also plotted cue characteristics in boxplots using the R package `ggplot2`, which displays the median, interquartile range (IQR), and whiskers, which represent the largest (or smallest) value above (or below) 1.5*IQR (Wickham, 2016). We plotted the proportion of cues classified as having or not having each content characteristic for each thinking condition. We also calculated the correlation between content characteristics at the cue-level for each thinking condition.

#### Cue content characteristics as a moderator of EFT

We used linear mixed effects models to predict delay discounting (ln(*k*)) using the interaction between thinking condition (EFT vs. not EFT) and each cue content characteristic (mean value across a participant’s cue set). One model was fit for each characteristic. We included a random intercept for study to account for variability explained by the different studies from which the data were generated. Models were conducted using the R package `lmerTest` (Kuznetsova et al., 2017) and 95% confidence intervals for model parameters were calculated using `stats::confint()`. For models with significant interaction terms, we conducted a simple slopes analysis using the R package `emtrends` to facilitate interpretation.

#### Model selection

We performed model selection to determine the optimal model for predicting delay discounting. We considered demographic variables, BMI, mean cue content characteristics, and interactions between each cue characteristic and thinking condition. All models included a random effect for study to account for study-level variability in delay discounting task parameters, population, and other factors. The optimal model was determined as that with the lowest Bayesian Information Criterion (BIC). Because of the large model space due to the total number of variables under consideration, a genetic algorithm was used to perform a stochastic search of the model space (Scrucca, 2013; J. Williams et al., 2023; Xu et al., 2023).

#### Principal components analysis (PCA)

In an exploratory analysis to understand the latent structure of the cue content characteristics, we performed PCA on the cue-level classifier predictions. Our goal was to identify constructs emerging from combinations of the cue content variables. All variables were scaled prior to PCA (i.e. using the correlation matrix) and eigenvalues and eigenvectors were determined. We identified the number of principal components (PCs) by selecting those PCs with eigenvalues greater than 1; that is the set of PCs that explain more variability than the mean variability in the data (i.e. Kaiser’s criterion; Supplementary Figure 2). Importantly, the number of PCs we considered does not impact the PC loadings (i.e. PC1 has the same loadings regardless of if 3 or 6 PCs are identified; Jolliffe & Cadima, 2016).

Next, we calculated the common factors by multiplying the eigenvectors (i.e. PC loadings) by the observed data, which henceforth will be referred to as *content domains (CD)*. Similar to other cue characteristics, we summarize the cues at the participant level using the mean CD value across a participant’s cue set. We then repeated the moderation analysis and model selection analysis with the calculated CDs.

## Results

### Participants

The total cohort used in this analysis was derived from 19 studies and resulted in 1705 unique individuals. Demographics for this total cohort was on average 40 years old, majority female (66%), not Hispanic (91.5%), and white (75%). 56% of the participants were assigned EFT, and the average ln(*k)* value after intervention was – 5.51 (ED50: 247 days). Table 2 breaks down demographics for participants with and without delay discounting data available (some participants had cues but studies did not measure delay discounting). The mean BMI of participants included in moderation analyses was 33.1. Study specific demographics are available in Supplementary Table 5.

**Table 2.** Participant characteristics for the total cohort and those in analyses with ln(*k*).

Variable	Measurement for ln(*k*)	
	No (*n* = 271)	Yes (*n* = 1434)
Age (mean (SD))	39.79 (8.98)	40.00 (13.58)
BMI (mean (SD))	32.65 (5.46)	33.16 (8.08)
Ethnicity (%)		
Hispanic	11 (4.1)	78 (5.4)
Not Hispanic	162 (59.8)	1313 (91.6)
NA	98 (36.2)	43 (3.0)
Gender (%)		
Female	175 (64.6)	887 (61.9)
Male	1 (0.4)	535 (37.3)
Non-binary/Other	0 (0.0)	3 (0.2)
NA	95 (35.1)	9 (0.6)
Household Income (mean (SD))	71,178.75 (69,299.29)	53,168.40 (42,863.59)
Education (%)		
High School or less	48 (17.7)	500 (34.9)
Some College, 2-year Degree, or Vocational Training	52 (19.2)	207 (14.4)
Completed a 4-year College Degree	9 (3.3)	165 (11.5)
Graduate or Professional Degree	67 (24.7)	518 (36.1)
NA	95 (35.1)	44 (3.1)
Race (%)		
American Indian or Alaska Native	1 (0.4)	10 (0.7)
Asian	2 (0.7)	69 (4.8)
Black or African American	42 (15.5)	162 (11.3)
Multi-racial	7 (2.6)	39 (2.7)
Native Hawaiian or Other Pacific Islander	0 (0.0)	5 (0.3)
Other race	1 (0.4)	23 (1.6)
White	123 (45.4)	1087 (75.8)
NA	95 (35.1)	39 (2.7)
Thinking Condition (%)		
EFT	153 (56.5)	814 (56.8)
Not EFT	118 (43.5)	620 (43.2)

### Cue content characteristics

**Figure 1.**
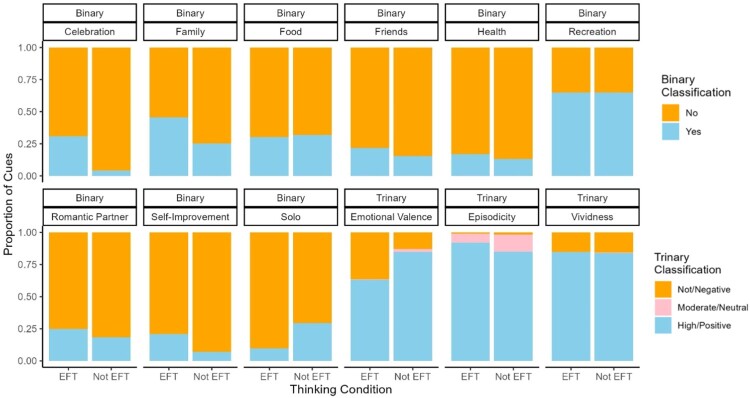
Cue Content Characteristics by Episodic Thinking Condition*.* Note. For all binary content areas, orange denotes the proportion of cues that were classified as not having a particular content characteristic and blue denotes the proportion of cues that were classified as having a particular content characteristic. For emotional valence, orange represents the proportion of cues classified as negative emotion, pink represents neutral emotion, and blue represents positive emotion. For episodicity and vividness, orange represents the proportion of cues classified as no or low levels, pink represents moderate levels, and blue represents high levels.

**Figure 2.**
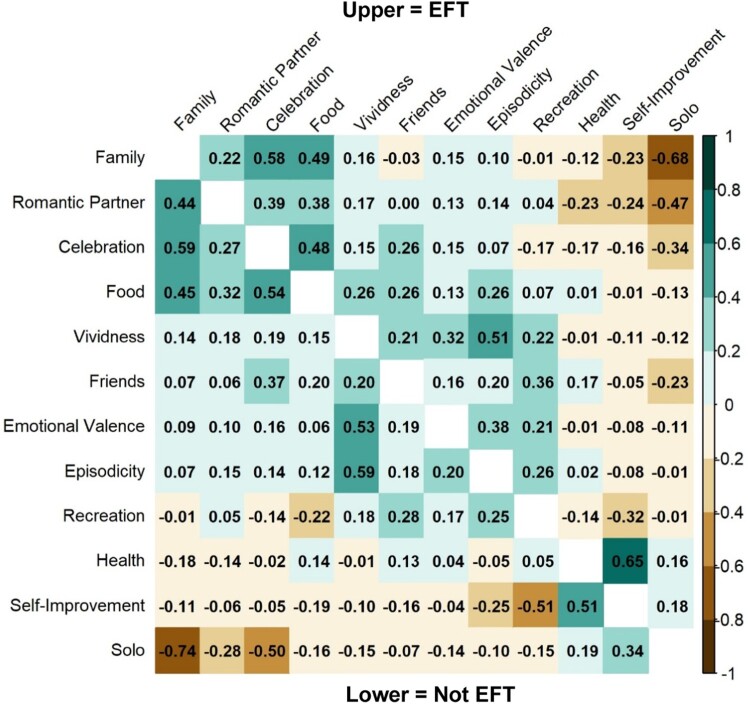
Correlations Between Cue Content Characteristics Split By Episodic Thinking Condition. Note. The upper correlation matrix is for EFT cues (*n* = 5760) and the lower correlation matrix is for Not EFT cues (*n* = 3428). Correlations are Spearman rank-order coefficients.

Figure 1 shows the proportion of cues classified as having or not having each content characteristic, by thinking condition. The proportion of cues predicted as having or not having each content area was similar for most content areas. However, Celebration, Self-Improvement, and Family appeared more frequently in EFT cues than Not EFT cues and Solo was more frequent in Not EFT cues. Figure 2 shows Spearman rank-order correlation coefficients for EFT and Not EFT cues. For both conditions, Solo was negatively associated with other person-related content (e.g. Family) and Celebration. In both conditions, Self-Improvement was positively associated with Health.

**Figure 3.**
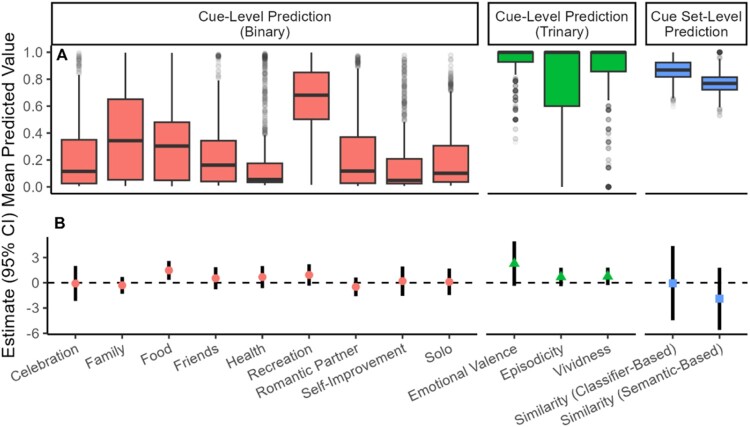
Cue Content Characteristics and Interactions Between Cue Content and Thinking Condition. Note. In the top panel (A), boxplots are for participants’ mean value across cues for NLP classifier predictions at the cue-level, and cue set-level values. In the bottom panel (B), points represent the estimated beta for the interaction term between the cue content characteristic and thinking condition in the mixed effects models predicting degree of delay discounting (ln(*k*)). Bars represent 95% confidence intervals.

Figure 3 shows the distribution of the mean predicted value (mean across a participant’s cue set) for each cue content characteristic. The mean predicted value was highest for Emotional Valence and Vividness (M = 0.89, SD = 0.23; M = 0.71, SD = 0.58). Intermediately, the mean predicted values for Family, Episodicity, and Recreation were 0.39, 0.49, and 0.66, respectively (SDs = 0.31, 0.7, 0.23). The mean predicted values were lower for Solo, Celebration, Friends, Romantic Partner, and Food (Ms = 0.2-0.32; SDs = 0.22-0.27). The mean values for Similarity (Classifier-Based and Semantic-Based) were 0.87 and 0.77, respectively (SDs = 0.08, 0.08). See Supplementary Table 6.

### Cue content characteristics as a moderator of intervention effect on delay discounting

**Figure 4.**
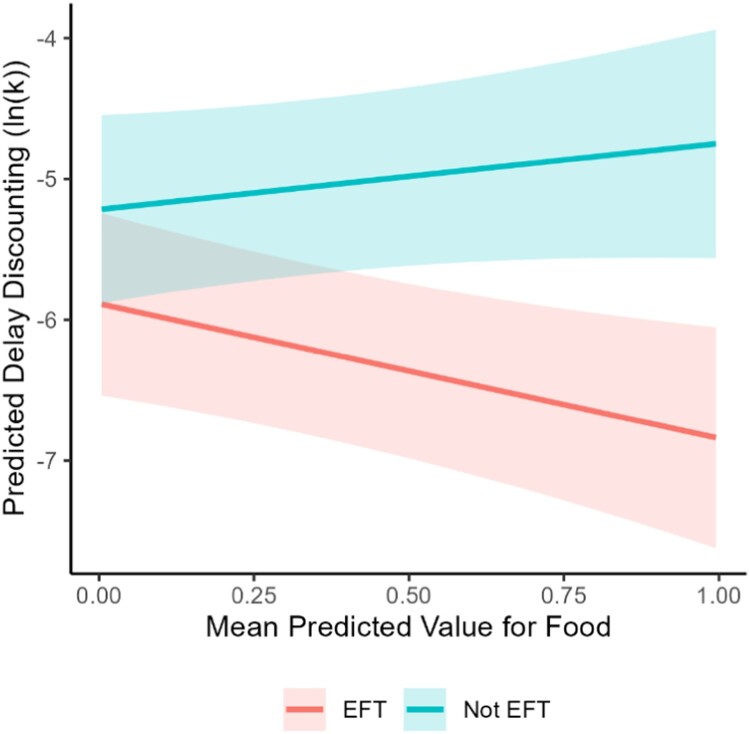
Interaction Between Food Cue Content Characteristic and Thinking Condition. Note. Red represents participants who engaged in episodic future thinking and blue represents participants who did not engage in episodic future thinking. Lines represent predictions from a linear mixed effects model and the shaded area represents confidence intervals.

To determine if the effect of thinking condition (EFT vs. control) on delay discounting differed based on cue content, in separate models, we tested all interactions between thinking condition and cue content variables. Of the 14 total cue content characteristics (see Figure 3), only the interaction between thinking condition and Food was significant (β = 1.48; 95% CI = 0.36, 2.6; *p* = 0.01; see Figure 4). In other words, for participants who were in the EFT condition, having more cues involving food (independent of other contextual factors) was associated with lower delay discounting (slope of – 0.983 in Figure 4) whereas for participants in a control condition, having more cues involving food was associated with higher delay discounting (slope of 0.36 in Figure 4). All other moderating roles of cue characteristics were not significant (βs from – 1.89–1.48; ps > =  0.09; see Figure 3 and Supplementary Table 7). See Supplementary Tables 8 and 9 for results when considering the minimum and maximum cue content characteristic values across a participant’s cue set.

### Model selection

We performed model selection to determine the optimal set of variables for predicting delay discounting. The optimal model includes education (*β *= −0.07342; *p* = .002848), race (White vs. Not White; *β *= −0.09428; *p* = .000147), thinking condition (*β* = −0.19113; *p* < 0.0001), body mass index (*β *= 0.08006; *p* = 0.003517) and vividness (*β *= −0.07949; *p* < .005320) as main effects and a random effect for study. No interaction terms were selected. We also considered models without the random effect, but these were excluded due to large BIC values (BIC_lm_ = 3980.832 vs. BIC_lmm_ = 3898.211). See Supplementary Table 10 for results when considering the minimum and maximum cue content characteristic values across a participant’s cue set (identical results).

### Cue content characteristic themes

**Figure 5.**
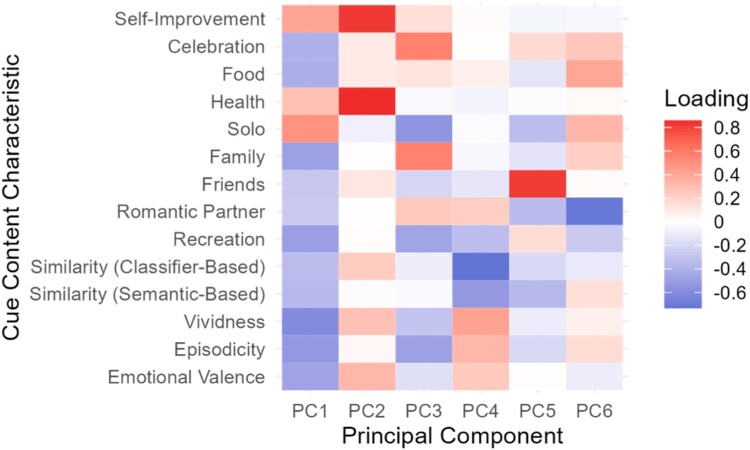
Principal Components Loadings. Note. Each column represents a principal component. Each row is a cue or cue-set level content characteristic. Darker cells indicate stronger loadings and lighter cells indicate weaker loadings. Blue represents negative loadings and red represents positive loadings.

We explored the presence of cue content characteristic themes through PCA. Six principal components were identified. The first domain had Vividness, Episodicity, and Recreation as the strongest contributors. The second domain had Health and Self-Improvement with the largest magnitude of factor loadings. The third domain included Celebration, Solo, and Family as primary contributors. Domain 4 primarily includes Similarity (Classifier-Based) and Similarity (Semantic-based). In Domain 5, Friends is the strongest contributor. Domain 6 includes Romantic Partner and Food. See Figure 5 for principal components loadings. An elbow plot and factor loading values can be found in Supplementary Materials (Supplementary Figure 2, Supplementary Table 11). These six dimensions explained 68.24% of the total variability in the data. The proportion of variation explained by each PC, respectively, was 18.49%, 12.16%, 11.92%, 9.7%, 8.6%, and 7.4%.

### Interaction between cue content themes and thinking condition

**Figure 6.**
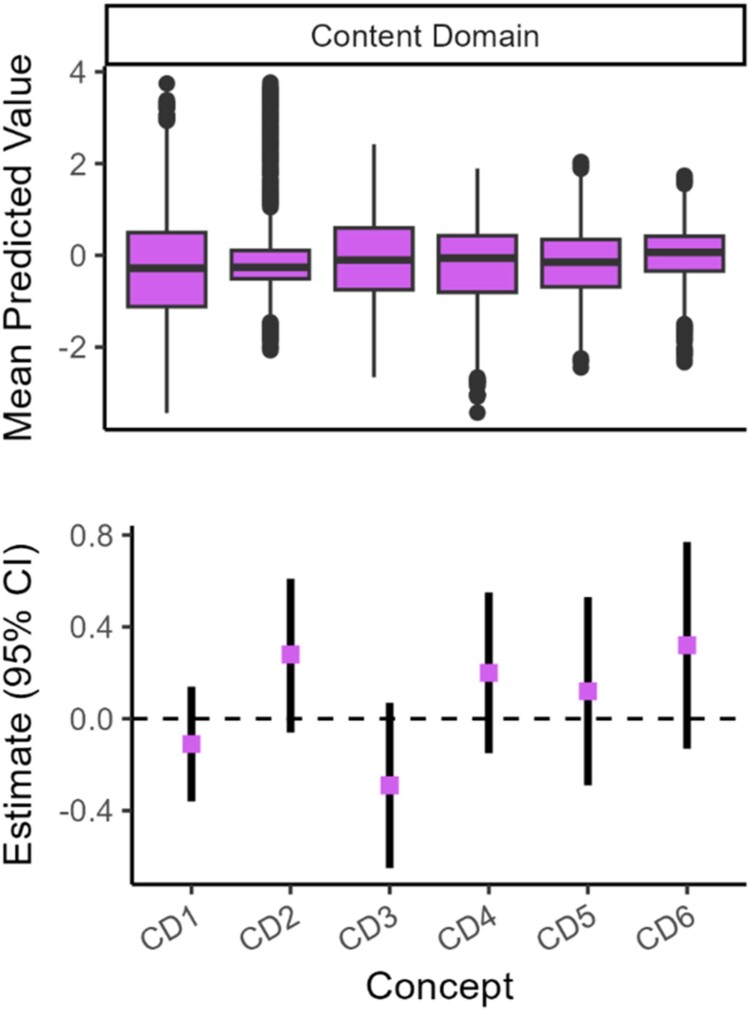
Principal Components from Cue Content Characteristics and Interactions Between Principal Components and Thinking Condition. Note. In the top panel (A), boxplots are for participants’ mean value across cues for principal components. In the bottom panel (B), points represent the estimated beta for the interaction term between the cue content characteristic and thinking condition in the mixed effects models predicting degree of delay discounting (ln(*k*)). Bars represent 95% confidence intervals.

We observed no significant interactions between principal components and thinking condition (*β*s from -0.35 to 0.28; *p*s from 0.11 to 0.62). See Figure 6 and Supplementary Table 12.

### Model selection including cue content domains

No content domains were included using model selection.

## Discussion

In this study, we described the content of cues from EFT studies primarily focused on samples with overweight/obesity using natural language processing classifier models. We also examined if cue content characteristics could moderate the impact of EFT on delay discounting. Food was a moderator of the effect of EFT on delay discounting in a model without adjusting for covariates, but no cue content areas were included as moderators in model selection. This may suggest that the moderating effect of food-related cues is attenuated when adjusting for covariates, covariates are confounded with the potential moderating effects of food-related cues, or that demographic covariates explain more variance in delay discounting than the potential moderating role of food-related cues. Given that we did not adjust for multiple comparisons in this exploratory study, we note that this result about Food requires further examination and should be interpreted with caution, especially since the model selection analysis did not replicate the findings of the interaction model. We also conducted principal components analysis to allow for interaction between multiple cue characteristics, finding 6 cue content domains. No content domains moderated the impact of EFT on delay discounting. Elaborating on the main analyses to consider other pattens of cue content by using minimum and maximum values instead of the mean, we similarly found no moderating effect of cue content in model selection analyses. In sum, we find little evidence that the cue characteristics we examined are associated with the impact of EFT on DD. Overall, however, these results are in contrast to our expectations and to earlier hypotheses about the potential benefit of having content about Health, Food, or Self-Improvement in EFT cues.

Note that all results should be interpreted in the context of how we defined each construct. Although our goal was to align content areas with previously studied constructs in the literature, consider that there may be slight variations in operationalization. Due to the nature of the data available (i.e. only what the participant has written), some constructs (e.g. cues that reflect an idealized self), may be difficult to study. We classified cues based on the text available, but it is important to consider that participants’ intent and understanding of the text and the event they imagined may differ from secondary evaluations of the text.

Overall, EFT appears to be similarly effective in its impact on delay discounting whether or not participants imagine the cue content areas we examined, such as Self-Improvement, Health, Family, and Celebration. These results may imply (especially for the binary cue characteristics) that we do not need to instruct participants to imagine about certain topics in their cues and that participants are welcome to imagine whatever events they choose in their EFT cues with little or no reduction in intervention efficacy. However, it is unknown if the effects of content on delay discounting are the same as the effect of content on other outcomes (e.g. behavioral economic demand). In addition, this study does not necessarily inform how EFT should be conducted with other populations. For instance, it may be important to ask participants with substance use to generate cues that do not include references to substance use (Chang & Ladd, 2023).

Factors to consider in interpreting the results of this study include the potential for ceiling effects and direction of causality. In all studies, in line with theory about how EFT works, participants were instructed to write vivid, positive, and episodic cues about future events. Thus, as reflected in Figure 1 and descriptive statistics, most participants’ cues were scored as having high levels of episodicity and vividness, and positive emotional valence. We expected that participants with more positive, vivid, and episodic cues would have larger changes in delay discounting. However, due to high levels for all of these areas, moderation analyses may be limited by ceiling effects or a restricted range. Nevertheless, these variables could still be important for effective EFT, but among participants who were instructed to include these qualities, we did not find evidence that they moderate the effect of EFT on delay discounting. Given the possible ceiling effects in this study and considering the available evidence, we would still recommend that participants be instructed to write positive, vivid, and episodic cues. Another note for interpretation in this study is that we examined cue content post-hoc. Therefore, it is important to consider confounding factors (e.g. participants interested in health behavior change may be more likely to spontaneously incorporate positive health behaviors into cues). Related, the effect of freely choosing to include some content area may be different than being instructed to include a content area.

There are some further limitations and considerations related to our NLP approach. One limitation is that although these NLP classifiers demonstrate acceptable accuracy, inevitably, some cues were misclassified (i.e. classification accuracy was lower than 100%). Lower accuracy in classifiers could make it harder to detect moderating effects of cue content areas, including episodicity and vividness. However, using NLP allowed us to examine for the first time the content of over 9000 cues, which is a strength and a novel contribution of this study. Future research could fine-tune NLP classifiers that showed trends toward moderation (e.g. Food) to increase their accuracy. One factor to consider is that we used a supervised machine learning approach to develop the classifiers, and developed classifiers specifically for content we were interested in, which may introduce some bias. Other approaches that do not rely on pre-specification (e.g. Latent Dirichlet Allocation) may identify other important areas. However, we used other NLP tools (e.g. text clustering/topic modeling, Semantic Search; Ahmadi et al., 2023) to identify content frequently described in cues (e.g. celebrations), which increases our confidence that highly prevalent content areas were described in this analysis. However, there may be cue content areas that we did not measure that are important for EFT efficacy. Despite this, we did examine 12 different cue-level content areas, many of which had been examined experimentally previously or had theoretical backing, further supporting the relative comprehensiveness of our classifiers. Note however that these analyses and classifiers primarily considered the impact of one content area at a time. Other approaches may be better suited to consider co-occurring content areas, and the effect of co-occurring concepts could have different effects. Future research could train additional NLP classifiers or use other NLP or large language model techniques (e.g. multi-label classification) to explore other content areas that could moderate the impact of EFT on DD. Another interesting future direction could be to conduct a subgroup analysis such as latent class or latent profile analysis.

Other considerations include the generalizability of the results. First, the data included in this study consisted only of studies conducted by three collaborating primary investigators. This may lead to some bias and may reduce generalizability. However, only a few other investigators have examined EFT in the context of obesity, so we captured a large proportion of this body of work. For instance, in a scoping review on EFT and overweight/obesity, 11 out of 16 studies included were conducted by at least one of the authors on this manuscript (Liu et al., 2023). Helping to mitigate this bias, using our own data allowed us to use individual-level measurements rather than means at the study level, which is a strength compared to traditional meta-analyses. In future studies, combining data from open science sources and increased collaboration among research teams would increase the data available to study and generalizability of findings. Next, our sample had a high mean BMI, and we did not include studies that purposefully recruited participants with high levels of substance use. Therefore, it is unknown if other populations would have the same cue content or if the moderating role of cue content is the same. Future work should characterize the content of cues generated by other populations. Still, our analysis dealt with delay discounting rather than heterogenous health behaviors across populations, potentially increasing generalizability.

### Conclusion and future directions

In this study, we used NLP classifiers to describe over 9000 cues from EFT studies that primarily consisted of people with overweight/obesity. To our knowledge, this is the largest and most systematic investigation of EFT cues. In general, we found that cues were emotionally positive, contained episodic details, and were highly vivid. We also found that participants tended to write about events that were with family more so than events with friends or romantic partners. Recreational events and events with food were discussed more than events involving celebrations, health, or self-improvement. Still, participants’ cue content varied substantially (see Figure 3).

In this study we also tested if cue content is important for the effect of EFT interventions on delay discounting. We conclude that EFT is similarly effective regardless of whether participants discuss the cue content areas we examined.

There are several future directions and implications resulting from this study. Other classifiers or NLP methods could be used to further characterize cues. For instance, within the Food content area, we were unable to distinguish between different types of foods or the context in which the food was prepared or eaten (e.g. intentionally preparing a healthy meal for dinner with family vs. indulging for a recreational experience). Thus, results of this study were unable to directly support or refute the content specificity hypothesis about EFT cue content, since the food-related behaviors may or may not have been related to positive health behavior change. Similarly, we only examined the emotional valence (positive to negative) of cues, whereas participants likely included several other more complex emotional descriptors (e.g. pride). For studies that use EFT, the present study implies that there is no additional benefit of instructing participants to imagine particular subjects. Still, the classifiers we developed could be used to characterize cues in future studies. For instance, classifiers could be used to increase confidence that participants followed cue generation instructions (e.g. inclusion of who is at the event, creating positive cues). Beyond the written word, we may learn more about EFT cue effectiveness by examining affect, facial expressions, and tone of voice while participants are imagining or repeating their cues.

In conclusion, we took a novel approach by applying NLP to systematically examine the content of over 9000 cues from EFT studies. This innovation may provide important insights to EFT mechanisms, efficacy, and implementation for modifying health behavior.

## Supplementary Material

supplementary materials revised r2.docx

## Data Availability

Data will be made available under reasonable request and consistent with the consent documents for each individual study.

## CRediT author statement

**Haylee Downey**: Conceptualization, Formal Analysis, Data curation, Writing – Original Draft, Writing – Review & Editing, Visualization. **Shuangshuang Xu:** Formal analysis, Data curation, Visualization. **Sareh Ahmadi**: Methodology, Writing – Original Draft. **Aditya Shah**: Methodology. **Jeremiah M. Brown**: Conceptualization, Writing – Review & Editing. **Warren K. Bickel**: Conceptualization, Resources. **Leonard H. Epstein:** Conceptualization, Writing – Review & Editing, Resources. **Allison N. Tegge:** Conceptualization, Formal analysis, Data Curation, Writing – Original Draft, Writing – Review & Editing, **Edward A. Fox:** Methodology, Resources, Writing – Review & Editing, Supervision. **Jeffrey S. Stein:** Conceptualization, Resources, Writing – Review & Editing, Supervision, Project administration, Funding acquisition.
