# Supplementary material for: What’s in a cue?: Using natural language processing to quantify content characteristics of episodic future thinking in the context of overweight and obesity
Source: Health Psychol Behav Med. 2025 Jun 2;13(1):2510417. doi: 10.1080/21642850.2025.2510417 (PMC12135091; doi:10.1080/21642850.2025.2510417)
Supplement: supplementary materials revised r2.docx [file RHPB_A_2510417_SM7840.docx]

What’s in a cue?: Using natural language processing to quantify content characteristics of episodic future thinking and better understand its use in the treatment of obesity

Supplementary Materials

**Supplementary Background**

**Development of Cue Content Classifiers**

A subset of our dataset was labeled by at least two different annotators, who were provided with category definitions (see Table 1) to guide their labeling. The data annotation process was conducted using Amazon Mechanical Turk and Qualtrics. We fine-tuned the RoBERTa model for a multi-label classifier on the labeled data, which included 2,058 training samples, 411 test samples, and 103 validation samples. The results, including accuracy, macro F1-score, precision, and recall, are presented in Table 2. These measures are based on true and false positives and negatives. For instance, a true positive is when the model correctly predicts a positive instance and a false negative is when the model incorrectly predicts a negative instance when it should be positive. Accuracy refers to the proportion of correctly classified data out of all the data predicted upon. Precision refers to how many of the predicted positive instances are actually correct. Recall (also called sensitivity or true positive rate) refers to the proportion of true positives were correctly identified. The F1 score combines precision and recall. For all metrics, higher values are better. See equations below:

$$Accuracy= \frac{TP+TN}{TP+TN+FP+FN}$$

$$Precision= \frac{TP}{TP+FP}$$

$$Recall= \frac{TP}{TP+FN}$$

$$F1= \frac{2 x Precision X Recall}{Precision+Recall}$$

| Table 1. |  |  |  |
| --- | --- | --- | --- |
| *Concept Descriptions and Example Cues* | | |  |
| Concept | Score | Definition | Example |
| Celebration | No | Contains no references to a celebration or a celebratory event. | In about 1 month I am fishing with my friends at Crater lake. I am with my friends Tyler and Dustin, sitting in my little boat. The sun is starting to set and I am having a great time catching some huge bass. |
|  | Yes | Contains an obvious, specific reference to a celebration or a celebratory event. Examples include but are not limited to graduations, birthdays, retirement parties, anniversaries, weddings, parties, and holidays such as Christmas and Thanksgiving. Look out for words such as "family", "birthday", "cake", "enjoying", "Christmas", "gifts", "presents", "celebrating", "proud", "son", "child", "graduation", "school", "wedding", "beautiful", and "friends", among others. | In about 6 months, I am **celebrating** my parent's **anniversary**. Rob and I take my parents out to dinner to **celebrate**. We go to a nice restaurant, and I am enjoying the atmosphere. It is just the four of us, married couples only. I am feeling good about being able to **celebrate this special day** with my parents. |
| Self-Improvement | No | Contains no references to "a better me", personal development, or self-improvement. | In about 3 years, I am relaxing with my wife at home. I am watching a movie with my wife and relaxing after a long day at work. I am so happy to have met someone that gets me like she does and I realize how lucky I am waking up by her side every day. |
|  | Yes | Contains obvious or specific references to "a better me", including personal development, self-improvement, making positive changes in life, achievements, hard work, or determination. May contain references to the idea that things are looking up or getting better. Look out for words such as "new", "house", "home", "car", "job", or "finally", among others. | In about 1 month, I am working full-time. I am sitting in my home office working for a company in a remote position. I am **relieved** that I have **found a job** at this time and that I can finally work on **getting my life back in order**. - In about 3 years, I am **finally** moving into my **new home**. My niece is helping me by making suggestions, and hiring helpers, since it is quite some time since I previously moved. I am very **excited about the change**. |
| Food | No | Contains no references to food, eating, or cooking. | In about 10 years, I am running an iron man after years and years of working out. I am finally in shape and ready to show off what I can do! |
|  | Yes | Contains obvious or specific references to food, eating, cooking, or a meal. Eating or food is a major and essential component of the text. | In 6 months I am **cooking dinner** for my family. I am over the hot stove while I prepare the **ham** to go into the over. As I do that I start water boiling for **macaroni and cheese**. As I am **cooking** my husband comes to me and he took a **bite of prepared food** I have sitting out and I joke with him about spoiling his **appetite**. As I get finished with **dinner** the kids, husband, and I gather at the kitchen table. We tell each other how much we all mean to one another and dig in. As I see my family I think of being lucky enough to have a family to enjoy this with. |
| Health | No | Contains no references to physical or mental health. Does not discuss physical state, mental health, or intentional changes in behaviors to improve health or health outcomes. | In about 6 months, I am at my daughters wedding. I am with my wife and we are having a great time. I am enjoying the venue in the mountains. |
|  | Yes | Contains an obvious, specific reference to physical or mental health. Examples include but are not limited to improved or worse physical state or mental health, intentional changes in behaviors to improve health (e.g., eating vegetables with dinner), and health outcomes (e.g., diabetes status or weight). Look out for words such as "weight", "pounds", "lost", "new", and "feel", among others. | In about 4 years, I am **jogging** 5 miles a day.  I am going **jogging** in the mornings and I love being able to enjoy the weather and get fresh air.  I am **going over hills like they are nothing.  I am breathing well and feel good and healthy**. |
| Recreation | No | Contains no references to engaging in an activity for leisure or fun while not working at one’s job. | In about 1 year, I am working at my new job. I am with my new coworkers who I haven't met yet. I am in the building where my employer is located. I am hard at work and trying to make a good impression. |
|  | Yes | Contains obvious or specific references to engaging in an activity for leisure or fun while not working at one’s job. Examples include but are not limited to sports or physical activities like running or hiking, art, movies and television, or hobbies like gardening. Look out for words such as "playing", "game", "enjoying", "beach", "ocean", "sand", "water", "hiking", "mountains", "trail", "park", and "trip", among others. | In about 3 years, I am **relaxing** with my wife at home. I am **watching a movie** with my wife and **relaxing after** a long day at work. I am so happy to have met someone that gets me like she does and I realize how lucky I am waking up by her side every day. |
| Episodic | Not at all | The writer primarily describes general knowledge of events or occurrences. The event is described as if the writer is not present or personally experiencing the event. | In about 5 years, my car is paid off. Sweeeet! Who doesn't love no more car payments? |
|  | Moderately | The writer describes both personal experiences, events, and actions in addition to general facts or ideas. The writer is somewhat in the moment, but also adds in a few facts or ideas. (Facts - italic, experiences - underline) | In 6 months, **I am visiting my mother in law.** *She is down visiting from Indiana. She is staying with us for a week.* *We have waited for her to come down for a long time because* *we have not seen her in years.* **We are ecstatic** she is here and is visiting. The kids are excited that they finally get to see her. **We are going shopping** and doing other fun things while she is here. **We are looking forward** to all the excitement that happens while she is visiting. |
|  | Highly | The writer primarily describes personal experiences, events, and actions, NOT general facts or ideas. The writer is describing events as if they are currently experiencing them "in the moment". The writer provides details about their own emotions and/or what they hear, see, or feel. | In about 10 years, **I am blowing** out the candles on my birthday cake and **feeling pleased** that my family has gathered because they love me so much. **I am smiling** because my adult children are making fun of how many candles are on my cake this year. **I hear** one of them jokingly say it's time to call the fire department and **everyone laughs** with love in their voices. |
| Vividness | Not at all | The text contains no details about the event. It is difficult to imagine the event. No context has been given regarding the event. | In about 5 years, I am in Antarctica for my birthday. |
|  | Moderately | The text contains only a few details or mostly non-specific details. The reader is left to fill in gaps, making it somewhat hard to imagine the event. More details could have been provided describing the event. Some context has been given regarding the event. | In about 3 months, I am going to the mall **with my husband**.  We am shopping **for a stove**.  I am excited. |
|  | Highly | The text contains sufficient and specific details so that the event described is readily and easily imaginable. A considerable amount of context has been given regarding the event. | In about 6 months, I am horseback riding in the **beautiful fall leaves.** I am **with my friend Sarah at Riverside Park**. I am riding my horse Dakota. The **trail is covered in Orange and yellow leaves**, and **they are falling** all around us. We are very happy and relaxed. It is nice and **cool out.** |
| Emotional Valence | Negative emotion | Primarily contains references to negative emotions or behaviors, including sadness, crying, or anger. | In about 3 months, I am going back to work and I'm **sad** because the holidays are over and I'm back to work |
|  | Neutral emotion | Contains references to both positive (e.g., laughing, smiling, or happiness) and  negative emotions or behaviors (e.g., crying, sadness); or contains weak or ambiguous references to positive or negative emotions and behaviors. | In about 5 years, I am going to watch my kids graduation. My son will walk across the stage my wife will be next to m. I will feel pride and **happiness**. But also be **sad** as my son will be moving on with his life. |
|  | Positive emotion | Primarily contains references to positive emotions or behaviors including laughing, smiling, and happiness. | In about 3 months, I am going to go to Florida. I feel so **happy** and **thrilled** to be here. The beaches are **great** and the weather is so warm**!** |

Table 2. *Classification Accuracy Metrics for Each Binary Cue Content Category*

| Category | Precision | Recall | F1-Score | Accuracy |
| --- | --- | --- | --- | --- |
| Health | 95 % | 98 % | 96 % | 97 % |
| Recreation | 84 % | 88 % | 86 % | 83 % |
| Self-improvement | 81 % | 69 % | 75 % | 88 % |
| Celebration | 95 % | 97 % | 96 % | 98 % |
| Food | 91 % | 97 % | 94 % | 96 % |
| Solo | 82 % | 76 % | 79 % | 93 % |
| Family | 95 % | 98 % | 96 % | 97 % |
| Romantic Partner | 88 % | 96 % | 92 % | 96 % |
| Friends | 91 % | 92 % | 92 % | 97 % |

*Note*: For all measures, higher values indicate greater quality.

For the emotional valence, vividness, and episodicity categories, each cue was annotated by at least two annotators. Each annotator assigned a score between 0 and 100 to the cue for each of the emotional valence, vividness, and episodicity categories. The final score for each cue was obtained by averaging these annotator-provided scores. For classification, the 0-100 range was divided into three classes: [0-33], [33-64], and [64-100], which correspond to the Not, Moderately, and Highly classes for each of these categories. This setup creates a multi-class classification framework, with each category containing three classes. To limit variability across annotator ratings, we considered only cues in which the range of scores was less than 15. We had 217, 198, and 411 labeled data samples for the vividness, episodicity, and emotional valence categories, respectively, available for building classifiers. Due to the small size of this labeled dataset, we leveraged few-shot learning using the FLAN-T5 11B large language model to optimize performance. The input prompt for the language model included definitions for each category, followed by a few labeled examples for few-shot in-context learning. Given the model's 2048-token limit, we could include up to 30 labeled samples for vividness, 21 for episodicity, and 18 for emotional valence. These few labeled data were randomly sampled from the available dataset, while the rest of the data was used for testing the classifier. The results are reported in Table 3.

Table 3. *Classification Accuracy Metrics for Each Trinary Cue Content Category*

| Category | Precision | Recall | F1-Score | Accuracy |
| --- | --- | --- | --- | --- |
| Vividness | 80 % | 71.6 % | 68 % | 82 % |
| Episodicity | 72 .6% | 72.6% | 65 % | 88 % |
| Emotional Valence | 77 % | 67 % | 70 % | 87 % |

*Note*: For all measures, higher values indicate greater quality.

**Rationale for NLP Approach and Identification of Content Characteristics**

Selection of concepts for content classifiers was iterative and data-based. In pilot work, we examined some pre-defined content areas that we had hypotheses about (e.g., health, episodicity), content characteristics examined elsewhere in the literature (e.g., health), and some exploratory/descriptive content areas. Because we had certain concepts we specifically wanted to characterize, we used the supervised learning approach described in the previous section rather than other approaches.

We initially collected annotation data using visual analog scales (VAS) for all content characteristics. However, ratings for most content characteristics using the VAS followed a bimodal distribution (with or without the content characteristic). Because of this, we switched to binary ratings for most content characteristics. In addition, in pilot work some content characteristics were infrequently observed (e.g., references to work/occupations), making analysis of these content characteristics difficult. To remedy the imbalanced data and identify more frequently occurring content, we applied other unsupervised natural language processing methods (e.g., text-based clustering) to identify frequent word pairs and clusters of cues. Then, from the topics and word pairs within these clusters, we derived the current content areas. For instance, celebration (i.e., of holidays and birthdays) was one area included using this approach.

**Supplementary Method**

**Measures**

The Similarity (Classifier-Based) measure is based on 11 binary classifiers. We did not report on 2 classifiers used in the Similarity (Classifier-Based) measure in the manuscript. The Pet classifier was designed to predict if the cue contained a reference to a pet. Too few cues were identified as containing references to pets that could be included in the Pet classifier training data, resulting in highly imbalanced data, so that classifier was not used. The Future classifier predicted if the cue was about a past event or a future event. As intended, the Future classifier was nearly perfectly correlated with thinking condition, so it was not appropriate to include this classifier in moderation analyses.

**Data Sources**

Athamneh, L. N., Stein, M. D., Lin, E. H., Stein, J. S., Mellis, A. M., Gatchalian, K. M., Epstein, L. H., & Bickel, W. K. (2021). Setting a goal could help you control: Comparing the effect of health goal versus general episodic future thinking on health behaviors among cigarette smokers and obese individuals. *Experimental and Clinical Psychopharmacology*, *29*(1), 59.

Bickel, W. K., Stein, J. S., Paluch, R. A., Mellis, A. M., Athamneh, L. N., Quattrin, T., Greenawald, M. H., Bree, K. A., Gatchalian, K. M., & Mastrandrea, L. D. (2020). Does episodic future thinking repair immediacy bias at home and in the laboratory in patients with prediabetes? *Psychosomatic medicine*, *82*(7), 699-707.

Brown, J. M., Bickel, W. K., Epstein, L. H., & Stein, J. S. (2023). Episodic future thinking in type 2 diabetes: Further development and validation of the Health Information Thinking control for clinical trials. *PLoS One*, *18*(8), e0289478.

Daniel, T. O., Stanton, C. M., & Epstein, L. H. (2013). The future is now: Comparing the effect of episodic future thinking on impulsivity in lean and obese individuals. *Appetite*, *71*, 120-125.

Epstein, L. H., Paluch, R. A., Biondolillo, M. J., Stein, J. S., Quattrin, T., Mastrandrea, L. D., Gatchalian, K., Greenawald, M. H., & Bickel, W. K. (2022). Effects of 6-month episodic future thinking training on delay discounting, weight loss and HbA1c changes in individuals with prediabetes. *Journal of Behavioral Medicine*, *45*(2), 227-239. https://doi.org/10.1007/s10865-021-00278-y

Hollis-Hansen, K., O’Donnell, S. E., Seidman, J. S., Brande, S. J., & Epstein, L. H. (2019). Improvements in episodic future thinking methodology: Establishing a standardized episodic thinking control. *PLoS One*, *14*(3), e0214397.

Hollis-Hansen, K., Seidman, J., O'Donnell, S., & Epstein, L. H. (2019). Episodic future thinking and grocery shopping online. *Appetite*, *133*, 1-9. https://doi.org/https://doi.org/10.1016/j.appet.2018.10.019

Hollis-Hansen, K., Seidman, J., O'Donnell, S., & Epstein, L. H. (2020). Mothers’ DASH diet adherence and food purchases after week-long episodic future thinking intervention. *Appetite*, *154*, 104757. https://doi.org/https://doi.org/10.1016/j.appet.2020.104757

Hollis-Hansen, K., Seidman, J., O'Donnell, S., Wedderburn, A., Stanar, S., Brande, S., & Epstein, L. H. (2020). An ecological momentary episodic future thinking intervention on mother's weekly food purchases. *Health Psychol*, *39*(2), 159-167. https://doi.org/10.1037/hea0000817

O'Donnell, S., Daniel, T. O., Koroschetz, J., Kilanowski, C., Otminski, A., Bickel, W. K., & Epstein, L. H. (2019). Do process simulations during episodic future thinking enhance the reduction of delay discounting for middle income participants and those living in poverty? *Journal of Behavioral Decision Making*, *32*(3), 231-240.

O'Neill, J., Daniel, T. O., & Epstein, L. H. (2016). Episodic future thinking reduces eating in a food court. *Eating Behaviors*, *20*, 9-13. https://doi.org/https://doi.org/10.1016/j.eatbeh.2015.10.002

O’Donnell, S., Hollis-Hansen, K., & Epstein, L. H. (2018). Mix and match: An investigation into whether episodic future thinking cues need to match discounting delays in order to be effective. *Behavioral Sciences*, *9*(1), 1.

Rung, J. M., & Madden, G. J. (2018). Experimental reductions of delay discounting and impulsive choice: A systematic review and meta-analysis. *Journal of experimental psychology: general*, *147*(9), 1349. https://doi.org/10.1037/xge0000462

Stein, J. S., Craft, W. H., Paluch, R. A., Gatchalian, K. M., Greenawald, M. H., Quattrin, T., Mastrandrea, L. D., Epstein, L. H., & Bickel, W. K. (2021). Bleak present, bright future: II. Combined effects of episodic future thinking and scarcity on delay discounting in adults at risk for type 2 diabetes. *Journal of Behavioral Medicine*, *44*(2), 222-230. https://doi.org/10.1007/s10865-020-00178-7

Stein, J. S., Sze, Y. Y., Athamneh, L., Koffarnus, M. N., Epstein, L. H., & Bickel, W. K. (2017). Think fast: rapid assessment of the effects of episodic future thinking on delay discounting in overweight/obese participants. *Journal of Behavioral Medicine*, *40*, 832-838. https://doi.org/10.1007/s10865-017-9857-8

Sze, Y. Y., Stein, J. S., Bickel, W. K., Paluch, R. A., & Epstein, L. H. (2017). Bleak present, bright future: Online episodic future thinking, scarcity, delay discounting, and food demand. *Clinical Psychological Science*, *5*(4), 683-697.

Vaughn, J. E., Ammermann, C., Lustberg, M. B., Bickel, W. K., & Stein, J. S. (2021). Delay discounting and adjuvant endocrine therapy adherence in hormone receptor-positive breast cancer. *Health Psychology*, *40*(6), 398. https://doi.org/10.1037/hea0001077

Supplementary Figure 1.

*Number and Timeframe of Cues in Each Study*


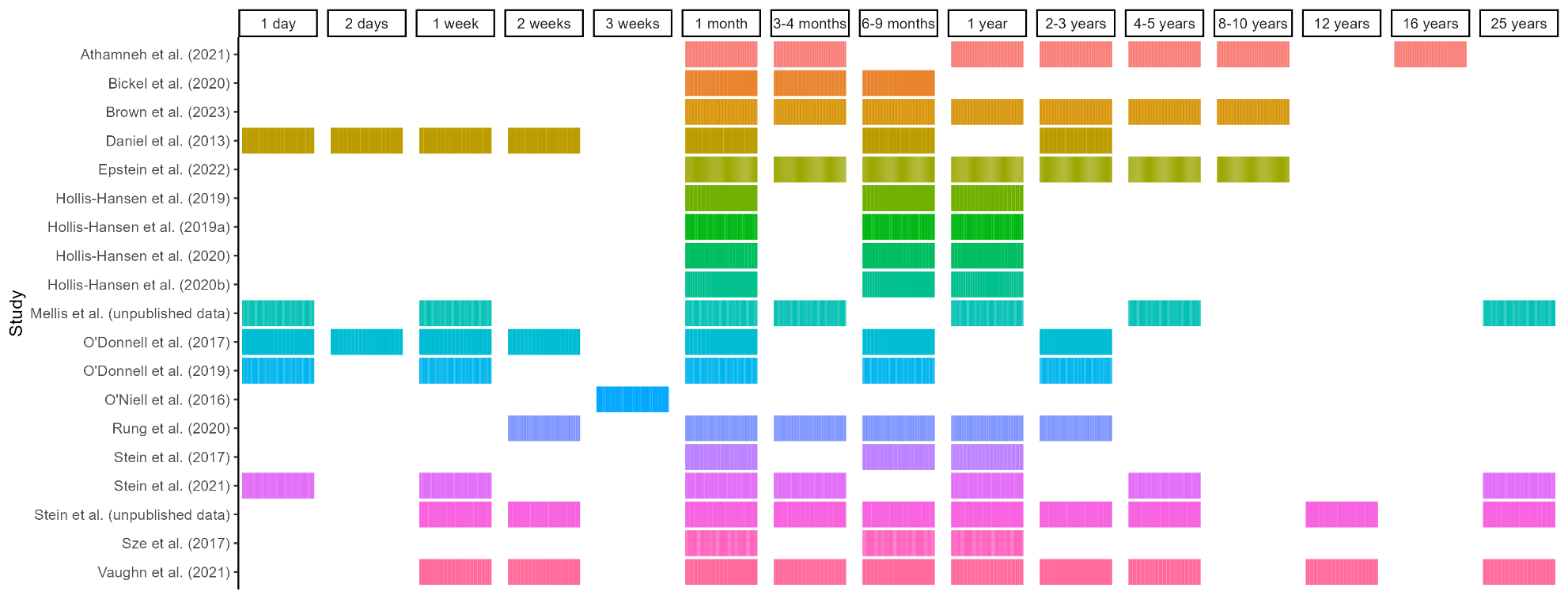


*Note.* Each row represents a study. The timeframe of the EFT cue in common units is on the x-axis. A colored block for a timeframe indicates that the study used that timeframe. The total number of colored blocks for a timeframe represented the number of cues in that study, except for in O’Neill et al. (2016) where all 3 cues used the same timeframe.

**Supplementary Results**

Supplementary Table 4.

*Study-Level Characteristics*

| Study | Population | Experimental conditions | Study type | LL Amount | Longest Delay |
| --- | --- | --- | --- | --- | --- |
| Athamneh et al. (2021) Exp. 2 | Overweight/obesity | EFT vs. ERT; health goal vs. general (four groups) | Online | 100 | 25 years |
| Bickel et al. (2020) | Prediabetes | EFT vs. ERT (two groups) | Lab | 1000 | 1 year |
| Brown et al. (2023) | T2DM w/obesity | EFT vs. HIT control (two groups) | Online | 1000 | 10 years |
| Daniel et al. (2013a) | Women with overweight/obesity | EFT vs. third-party narratives (two groups) | Lab | 100 | 2 years |
| Epstein et al. (2022) | Prediabetes | EFT vs. DCI (two groups) | Clinical | 100 | 25 years |
| Hollis-Hansen et al. (2019a) | General population | EFT vs. ERT vs SET | Lab | 100 | 2 years |
| Hollis-Hansen et al. (2019b) | Mothers with overweight/obesity | Study 1: EFT vs. money saving control; Study 2: Goal related EFT vs. general EFT vs. ERT | Lab | — | — |
| Hollis-Hansen et al. (2020a) | Mothers with overweight/obesity | EFT vs. SET (two groups) | Field | 1000 | 1 year |
| Hollis-Hansen et al. (2020b) | Mothers with overweight/obesity | EFT vs. SET; DASH education vs Safety education (four groups) | Field | — | — |
| Unpublished data (Mellis et al.) | Overweight/obesity | EFT vs. ERT | Online | 100 | 25 years |
| O'Donnell et al. (2018) Exp. 2 | People with lower income | EFT vs. ERT; process vs. non-process | Lab | 100 | 2 years |
| O'Donnell et al. (2019) | General population | EFT vs. ERT; cues matched vs. unmatched to DD delays (four groups) | Online | 100 | 2 years |
| O'Niell et al. (2016) | Women with overweight/obesity | EFT vs. ERT | Field | — | — |
| Rung & Epstein (2020) | General population | EFT vs. ERT vs. HIT control | Online | 100 | 2 years |
| Stein et al. (2017) | Overweight/obesity | EFT vs. ERT; 1 vs. 3 cue (4 groups) | Online | 100 | 25 years |
| Stein et al. (2021) | Prediabetes with overweight/obesity | EFT vs. ERT; scarcity vs. neutral narrative (four groups) | Lab | 100 | 25 years |
| Unpublished data; NCT03732209 (Stein et. al) | T2DM with obesity | EFT vs. DCI | Clinical | 100 | 1 year |
| Sze et al. (2017) Exp. 2 | Overweight/obesity | EFT vs. ERT vs. Control; scarcity narrative vs neutral narrative (6 groups) | Online | 100 | 1 year |
| Vaughn et al. (2021) | Breast cancer survivors with overweight/obesity | EFT vs. ERT (two groups) | Lab | 100 | N/A |

Supplementary Table 5.

*Participant Characteristics by Study*

|  | Study | | | | | | | | | | | | | | | | | | |
| --- | --- | --- | --- | --- | --- | --- | --- | --- | --- | --- | --- | --- | --- | --- | --- | --- | --- | --- | --- |
|  | Athamneh et al. (2021) | Bickel et al. (2020) | Brown et al. (2023) | Daniel et al. (2013) | Epstein et al. (2022) | Hollis-Hansen et al. (2019a) | Hollis-Hansen et al. (2019b) | Hollis-Hansen et al. (2020a) | Hollis-Hansen et al. (2020b) | Unpublished data (Mellis et al.) | O'Donnell et al. (2018) | O'Donnell et al. (2019) | O'Niell et al. (2016) | Rung et al. (2020) | Stein et al. (2017) | Stein et al. (2021) | Unpublished data; NCT03732209 (Stein et al.) | Sze et al. (2017) | Vaughn et al. (2021) |
| n | 262 | 69 | 113 | 31 | 28 | 52 | 81 | 42 | 60 | 51 | 36 | 160 | 34 | 133 | 141 | 78 | 7 | 166 | 67 |
| Age (mean (SD)) | 36.49 (11.02) | 60.19 (11.86) | 44.77 (12.12) | 24.14 (4.09) | 55.39 (8.45) | 29.19 (8.49) | 41.72 (8.37) | 38.05 (5.35) | 39.20 (5.69) | 35.06 (8.52) | 38.44 (10.82) | 34.23 (9.42) | 36.72 (13.14) | 37.80 (12.30) | 35.65 (10.70) | 50.85 (12.76) | 51.43 (9.16) | 37.75 (11.82) | 59.27 (9.27) |
| BMI (mean (SD)) | 36.77 (6.57) | 34.44 (7.96) | 39.04 (7.97) | 24.17 (3.49) | 33.35 (7.58) | 28.19 (7.04) | — | 35.94 (7.43) | 33.19 (5.91) | 34.81 (5.10) | 33.12 (10.27) | 27.36 (7.40) | 31.59 (4.51) | 26.78 (7.21) | 32.44 (5.92) | 37.50 (8.42) | — | 33.73 (6.70) | 32.57 (5.69) |
| Ethnicity (%) | |  |  |  |  |  |  |  |  |  |  |  |  |  |  |  |  |  |  |
| Hispanic | 19 (7.3) | 1 (1.4) | 14 (12.4) | 0 (0.0) | 2 (7.1) | 1 (1.9) | 6 (7.4) | 0 (0.0) | 4 (6.7) | 5 (9.8) | 2 (5.6) | 9 (5.6) | 1 (2.9) | 6 (4.5) | 5 (3.5) | 2 (2.6) | 1 (14.3) | 11 (6.6) | 0 (0.0) |
| Not Hispanic | 240 (91.6) | 68 (98.6) | 99 (87.6) | 0 (0.0) | 26 (92.9) | 51 (98.1) | 72 (88.9) | 42 (100.0) | 56 (93.3) | 44 (86.3) | 33 (91.7) | 150 (93.8) | 33 (97.1) | 127 (95.5) | 136 (96.5) | 71 (91.0) | 6 (85.7) | 155 (93.4) | 67 (100.0) |
| NA | 3 (1.1) | 0 (0.0) | 0 (0.0) | 31 (100.0) | 0 (0.0) | 0 (0.0) | 3 (3.7) | 0 (0.0) | 0 (0.0) | 2 (3.9) | 1 (2.8) | 1 (0.6) | 0 (0.0) | 0 (0.0) | 0 (0.0) | 5 (6.4) | 0 (0.0) | 0 (0.0) | 0 (0.0) |
| Gender (%) | |  |  |  |  |  |  |  |  |  |  |  |  |  |  |  |  |  |  |
| Female | 155 (59.2) | 40 (58.0) | 64 (56.6) | 31 (100.0) | 24 (85.7) | 36 (69.2) | 80 (98.8) | 42 (100.0) | 60 (100.0) | 26 (51.0) | 32 (88.9) | 76 (47.5) | 34 (100.0) | 69 (51.9) | 79 (56.0) | 56 (71.8) | 3 (42.9) | 89 (53.6) | 67 (100.0) |
| Male | 105 (40.1) | 29 (42.0) | 48 (42.5) | 0 (0.0) | 4 (14.3) | 16 (30.8) | 1 (1.2) | 0 (0.0) | 0 (0.0) | 24 (47.1) | 4 (11.1) | 84 (52.5) | 0 (0.0) | 61 (45.9) | 61 (43.3) | 18 (23.1) | 4 (57.1) | 77 (46.4) | 0 (0.0) |
| Non-binary/Other | 0 (0.0) | 0 (0.0) | 1 (0.9) | 0 (0.0) | 0 (0.0) | 0 (0.0) | 0 (0.0) | 0 (0.0) | 0 (0.0) | 0 (0.0) | 0 (0.0) | 0 (0.0) | 0 (0.0) | 2 (1.5) | 0 (0.0) | 0 (0.0) | 0 (0.0) | 0 (0.0) | 0 (0.0) |
| NA | 2 (0.8) | 0 (0.0) | 0 (0.0) | 0 (0.0) | 0 (0.0) | 0 (0.0) | 0 (0.0) | 0 (0.0) | 0 (0.0) | 1 (2.0) | 0 (0.0) | 0 (0.0) | 0 (0.0) | 1 (0.8) | 1 (0.7) | 4 (5.1) | 0 (0.0) | 0 (0.0) | 0 (0.0) |
| Household Income (mean (SD)) | 59007.15 (38870.20) | 82500.00 (55727.51) | 38188.69 (31998.16) | — | 36800.00 (27075.51) | 60884.62 (47493.15) | 75616.79 (42627.62) | 73190.32 (42934.44) | 86045.48 (100442.98) | 56121.95 (32823.16) | 25624.81 (14310.24) | 47101.42 (35423.87) | 32539.85 (26698.05) | 47053.83 (53534.47) | 34093.70 (27817.42) | 49452.64 (44168.90) | 74999.50 (37080.99) | 54382.22 (37502.65) | 88582.09 (59612.42) |
| Education (%) | |  |  |  |  |  |  |  |  |  |  |  |  |  |  |  |  |  |  |
| High School or less | 110 (42.0) | 20 (29.0) | 36 (31.9) | 0 (0.0) | 10 (35.7) | 17 (32.7) | 18 (22.2) | 16 (38.1) | 20 (33.3) | 20 (39.2) | 5 (13.9) | 78 (48.8) | 10 (29.4) | 56 (42.1) | 44 (31.2) | 12 (15.4) | 0 (0.0) | 60 (36.1) | 16 (23.9) |
| Some College, 2-year Degree, or Vocational Training | 34 (13.0) | 21 (30.4) | 15 (13.3) | 0 (0.0) | 7 (25.0) | 6 (11.5) | 28 (34.6) | 15 (35.7) | 17 (28.3) | 5 (9.8) | 2 (5.6) | 15 (9.4) | 7 (20.6) | 15 (11.3) | 16 (11.3) | 16 (20.5) | 2 (28.6) | 22 (13.3) | 16 (23.9) |
| Completed a 4-year College Degree | 19 (7.3) | 4 (5.8) | 7 (6.2) | 0 (0.0) | 1 (3.6) | 9 (17.3) | 4 (4.9) | 3 (7.1) | 3 (5.0) | 3 (5.9) | 15 (41.7) | 13 (8.1) | 1 (2.9) | 37 (27.8) | 16 (11.3) | 10 (12.8) | 2 (28.6) | 17 (10.2) | 10 (14.9) |
| Graduate or Professional Degree | 99 (37.8) | 19 (27.5) | 55 (48.7) | 0 (0.0) | 10 (35.7) | 20 (38.5) | 31 (38.3) | 8 (19.0) | 20 (33.3) | 23 (45.1) | 14 (38.9) | 54 (33.8) | 16 (47.1) | 23 (17.3) | 65 (46.1) | 34 (43.6) | 3 (42.9) | 67 (40.4) | 25 (37.3) |
| NA | 0 (0.0) | 5 (7.2) | 0 (0.0) | 31 (100.0) | 0 (0.0) | 0 (0.0) | 0 (0.0) | 0 (0.0) | 0 (0.0) | 0 (0.0) | 0 (0.0) | 0 (0.0) | 0 (0.0) | 2 (1.5) | 0 (0.0) | 6 (7.7) | 0 (0.0) | 0 (0.0) | 0 (0.0) |
| Race (%) |  |  |  |  |  |  |  |  |  |  |  |  |  |  |  |  |  |  |  |
| American Indian or Alaska Native | 3 (1.1) | 0 (0.0) | 1 (0.9) | 0 (0.0) | 0 (0.0) | 0 (0.0) | 1 (1.2) | 0 (0.0) | 0 (0.0) | 0 (0.0) | 0 (0.0) | 2 (1.2) | 0 (0.0) | 1 (0.8) | 1 (0.7) | 2 (2.6) | 0 (0.0) | 0 (0.0) | 0 (0.0) |
| Asian | 9 (3.4) | 0 (0.0) | 5 (4.4) | 0 (0.0) | 0 (0.0) | 12 (23.1) | 0 (0.0) | 0 (0.0) | 0 (0.0) | 2 (3.9) | 2 (5.6) | 22 (13.8) | 2 (5.9) | 6 (4.5) | 7 (5.0) | 1 (1.3) | 0 (0.0) | 3 (1.8) | 0 (0.0) |
| Black or African American | 24 (9.2) | 6 (8.7) | 11 (9.7) | 0 (0.0) | 6 (21.4) | 10 (19.2) | 21 (25.9) | 9 (21.4) | 16 (26.7) | 9 (17.6) | 10 (27.8) | 12 (7.5) | 5 (14.7) | 11 (8.3) | 11 (7.8) | 27 (34.6) | 1 (14.3) | 14 (8.4) | 2 (3.0) |
| Multi-racial | 8 (3.1) | 0 (0.0) | 3 (2.7) | 0 (0.0) | 1 (3.6) | 0 (0.0) | 4 (4.9) | 0 (0.0) | 0 (0.0) | 2 (3.9) | 3 (8.3) | 5 (3.1) | 3 (8.8) | 5 (3.8) | 3 (2.1) | 1 (1.3) | 0 (0.0) | 6 (3.6) | 2 (3.0) |
| Native Hawaiian or Other Pacific Islander | 1 (0.4) | 0 (0.0) | 1 (0.9) | 0 (0.0) | 0 (0.0) | 1 (1.9) | 0 (0.0) | 0 (0.0) | 0 (0.0) | 0 (0.0) | 0 (0.0) | 1 (0.6) | 0 (0.0) | 0 (0.0) | 1 (0.7) | 0 (0.0) | 0 (0.0) | 0 (0.0) | 0 (0.0) |
| Other race | 4 (1.5) | 2 (2.9) | 2 (1.8) | 0 (0.0) | 2 (7.1) | 1 (1.9) | 1 (1.2) | 0 (0.0) | 0 (0.0) | 1 (2.0) | 0 (0.0) | 0 (0.0) | 0 (0.0) | 4 (3.0) | 2 (1.4) | 2 (2.6) | 1 (14.3) | 2 (1.2) | 0 (0.0) |
| White | 212 (80.9) | 61 (88.4) | 90 (79.6) | 0 (0.0) | 19 (67.9) | 28 (53.8) | 54 (66.7) | 33 (78.6) | 44 (73.3) | 37 (72.5) | 20 (55.6) | 117 (73.1) | 24 (70.6) | 106 (79.7) | 116 (82.3) | 41 (52.6) | 5 (71.4) | 140 (84.3) | 63 (94.0) |
| NA | 1 (0.4) | 0 (0.0) | 0 (0.0) | 31 (100.0) | 0 (0.0) | 0 (0.0) | 0 (0.0) | 0 (0.0) | 0 (0.0) | 0 (0.0) | 1 (2.8) | 1 (0.6) | 0 (0.0) | 0 (0.0) | 0 (0.0) | 4 (5.1) | 0 (0.0) | 1 (0.6) | 0 (0.0) |
| Thinking Condition (%) | | |  |  |  |  |  |  |  |  |  |  |  |  |  |  |  |  |  |
| EFT | 130 (49.6) | 36 (52.2) | 113 (100.0) | 17 (54.8) | 28 (100.0) | 18 (34.6) | 48 (59.3) | 23 (54.8) | 30 (50.0) | 51 (100.0) | 18 (50.0) | 80 (50.0) | 16 (47.1) | 62 (46.6) | 72 (51.1) | 41 (52.6) | 7 (100.0) | 84 (50.6) | 36 (53.7) |
| Not EFT | 132 (50.4) | 33 (47.8) | 0 (0.0) | 14 (45.2) | 0 (0.0) | 34 (65.4) | 33 (40.7) | 19 (45.2) | 30 (50.0) | 0 (0.0) | 18 (50.0) | 80 (50.0) | 18 (52.9) | 71 (53.4) | 69 (48.9) | 37 (47.4) | 0 (0.0) | 82 (49.4) | 31 (46.3) |
| NA | 0 (0.0) | 0 (0.0) | 0 (0.0) | 0 (0.0) | 0 (0.0) | 0 (0.0) | 0 (0.0) | 0 (0.0) | 0 (0.0) | 0 (0.0) | 0 (0.0) | 0 (0.0) | 0 (0.0) | 0 (0.0) | 0 (0.0) | 0 (0.0) | 0 (0.0) | 0 (0.0) | 0 (0.0) |
| ln(k) (mean (SD)) | -4.20 (3.73) | -7.12 (2.56) | -7.04 (2.22) | -7.57 (2.74) | -8.77 (4.50) | -4.07 (1.82) | — | -5.64 (2.89) | — | -5.62 (3.52) | -5.33 (3.54) | -6.00 (2.22) | — | -5.47 (2.07) | -5.26 (2.29) | -5.04 (2.18) | -4.33 (4.13) | -5.55 (2.51) | -5.07 (1.74) |

Supplementary Table 6.

*Cue-Set Level Content Characteristics*

|  | Measurement for ln(k) | |
| --- | --- | --- |
| Variable (Mean (SD)) | No (n = 271) | Yes (n = 1435) |
| Family | 0.38 (0.36) | 0.39 (0.30) |
| Friends | 0.18 (0.21) | 0.23 (0.22) |
| Romantic Partner | 0.16 (0.21) | 0.24 (0.26) |
| Solo | 0.25 (0.27) | 0.19 (0.20) |
| Health | 0.25 (0.33) | 0.15 (0.22) |
| Recreation | 0.69 (0.27) | 0.65 (0.22) |
| Celebration | 0.22 (0.25) | 0.21 (0.23) |
| Self-Improvement | 0.20 (0.27) | 0.15 (0.20) |
| Food | 0.27 (0.31) | 0.33 (0.26) |
| Vividness | 0.64 (0.71) | 0.73 (0.56) |
| Episodicity | 0.33 (0.83) | 0.51 (0.67) |
| Emotional Valence | 0.88 (0.28) | 0.89 (0.22) |
| Similarity (Classifier-Based) | 0.91 (0.08) | 0.86 (0.08) |
| Similarity (Semantic-Based) | 0.91 (0.08) | 0.86 (0.08) |

Supplementary Table 7.

*Mean Cue Content Characteristics as a Moderator of the Effect of Thinking Condition on Delay Discounting (ln(k))*

| Variable in interaction with Thinking Condition | β | Standard Error | CI - lower | CI - upper | p-value |
| --- | --- | --- | --- | --- | --- |
| Family | -0.3 | 0.51 | -1.31 | 0.7 | 0.558 |
| Friends | 0.54 | 0.67 | -0.78 | 1.85 | 0.427 |
| Romantic Partner | -0.48 | 0.57 | -1.59 | 0.63 | 0.397 |
| Solo | 0.11 | 0.8 | -1.46 | 1.69 | 0.89 |
| Health | 0.68 | 0.67 | -0.63 | 1.99 | 0.309 |
| Recreation | 0.93 | 0.65 | -0.34 | 2.2 | 0.154 |
| Celebration | -0.08 | 1.06 | -2.16 | 2 | 0.939 |
| Self-Improvement | 0.2 | 0.89 | -1.55 | 1.95 | 0.824 |
| Food | 1.48 | 0.57 | 0.36 | 2.6 | 0.01 |
| Vividness | 0.76 | 0.53 | -0.28 | 1.8 | 0.151 |
| Episodicity | 0.68 | 0.56 | -0.41 | 1.79 | 0.224 |
| Emotional Valence | 2.28 | 1.35 | -0.36 | 4.93 | 0.091 |
| Similarity (Classifier-Based) | -0.06 | 2.26 | -4.46 | 4.38 | 0.979 |
| Similarity (Semantic-Based) | -1.89 | 1.89 | -5.6 | 1.79 | 0.316 |

Supplementary Table 8.

*Minimum Cue Content Characteristics as a Moderator of the Effect of Thinking Condition on Delay Discounting (ln(k))*

| Variable in interaction | β | Standard Error | CI - lower | CI - upper | p-value |
| --- | --- | --- | --- | --- | --- |
| Family | -0.3 | 0.63 | -1.54 | 0.93 | 0.633 |
| Friends | 0.11 | 1 | -1.85 | 2.07 | 0.911 |
| Romantic Partner | -0.2 | 0.78 | -1.74 | 1.33 | 0.796 |
| Solo | -0.29 | 1.46 | -3.14 | 2.57 | 0.844 |
| Health | 1.66 | 0.96 | -0.22 | 3.53 | 0.083 |
| Recreation | 0.02 | 0.44 | -0.84 | 0.87 | 0.967 |
| Celebration | -2.61 | 1.62 | -5.78 | 0.56 | 0.107 |
| Self-Improvement | 1.42 | 1.68 | -1.87 | 4.7 | 0.398 |
| Food | 0.3 | 0.77 | -1.22 | 1.81 | 0.703 |
| Vividness | -0.13 | 0.32 | -0.76 | 0.5 | 0.695 |
| Episodicity | 0.05 | 0.31 | -0.56 | 0.67 | 0.861 |
| Emotional Valence | 0.55 | 0.5 | -0.43 | 1.54 | 0.272 |
| Similarity (Classifier-Based) | -0.06 | 2.26 | -4.46 | 4.38 | 0.979 |
| Similarity (Semantic-Based) | -1.89 | 1.89 | -5.6 | 1.79 | 0.316 |

Supplementary Table 9.

*Maximum Cue Content Characteristics as a Moderator of the Effect of Thinking Condition on Delay Discounting (ln(k))*

| Variable in interaction | β | Standard Error | CI - lower | CI - upper | p-value |
| --- | --- | --- | --- | --- | --- |
| Family | 0.22 | 0.39 | -0.55 | 0.98 | 0.578 |
| Friends | 0.45 | 0.35 | -0.24 | 1.13 | 0.204 |
| Romantic Partner | -0.21 | 0.34 | -0.88 | 0.45 | 0.531 |
| Solo | 0.37 | 0.4 | -0.41 | 1.16 | 0.35 |
| Health | -0.27 | 0.38 | -1.02 | 0.49 | 0.486 |
| Recreation | -0.16 | 1.03 | -2.18 | 1.87 | 0.877 |
| Celebration | 0.5 | 0.45 | -0.39 | 1.39 | 0.271 |
| Self-Improvement | -0.34 | 0.43 | -1.17 | 0.5 | 0.427 |
| Food | 1.21 | 0.38 | 0.47 | 1.95 | 0.001 |
| Vividness | 1.47 | 0.61 | 0.27 | 2.67 | 0.016 |
| Episodicity | 2.07 | 0.73 | 0.65 | 3.51 | 0.005 |
| Emotional Valence | 0.18 | 3.08 | -5.86 | 6.22 | 0.954 |
| Similarity (Classifier-Based) | -0.12 | 2.26 | -4.52 | 4.33 | 0.959 |
| Similarity (Semantic-Based) | -1.88 | 1.89 | -5.59 | 1.8 | 0.319 |

Supplementary Table 10.

*Model Selection for Mixed Effects Models with Minimum or Maximum Cue Content Characteristics Predicting Delay Discounting (ln(k))*

| Variable | β | Standard Error | CI - lower | CI - upper | p-value |
| --- | --- | --- | --- | --- | --- |
| Intercept | -0.07 | 0.09 | -0.25 | 0.1 | 0.42 |
| Body Mass Index | 0.08 | 0.03 | 0.03 | 0.13 | 0.003 |
| Education | -0.07 | 0.02 | -0.12 | -0.03 | 0.002 |
| Race - White | -0.1 | 0.02 | -0.15 | -0.05 | <0.001 |
| Thinking Condition | -0.19 | 0.02 | -0.24 | -0.14 | <0.001 |

Supplementary Figure 2.

*Elbow Plot of Eigenvalues for Principal Components Analysis*


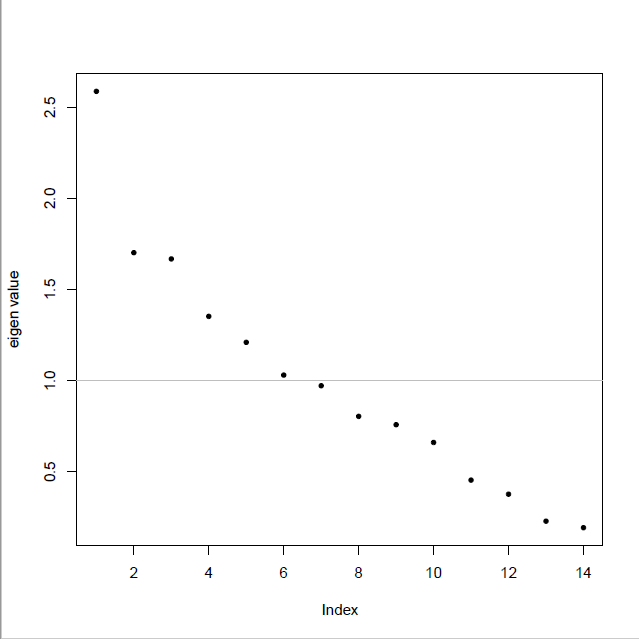


Supplementary Table 11.

*Principal Component Loadings*

|  | PC1 | PC2 | PC3 | PC4 | PC5 | PC6 |
| --- | --- | --- | --- | --- | --- | --- |
| Self-Improvement | 0.41 | 0.83 | 0.15 | 0.02 | -0.05 | -0.04 |
| Celebration | -0.4 | 0.1 | 0.57 | 0.01 | 0.17 | 0.26 |
| Food | -0.42 | 0.09 | 0.13 | 0.07 | -0.13 | 0.4 |
| Health | 0.28 | 0.86 | -0.03 | -0.06 | -0.02 | 0.03 |
| Solo | 0.5 | -0.08 | -0.55 | -0.02 | -0.33 | 0.34 |
| Family | -0.49 | 0.01 | 0.57 | -0.04 | -0.14 | 0.22 |
| Friends | -0.29 | 0.11 | -0.2 | -0.13 | 0.82 | 0.02 |
| Romantic Partner | -0.27 | -0.01 | 0.25 | 0.23 | -0.35 | -0.71 |
| Recreation | -0.5 | 0.01 | -0.46 | -0.33 | 0.15 | -0.27 |
| Similarity (Classifier-Based) | -0.34 | 0.23 | -0.1 | -0.73 | -0.19 | -0.11 |
| Similarity (Semantic-Based) | -0.36 | -0.01 | -0.03 | -0.53 | -0.36 | 0.14 |
| Vividness | -0.6 | 0.28 | -0.3 | 0.42 | -0.09 | 0.07 |
| Episodicity | -0.53 | 0.04 | -0.49 | 0.33 | -0.2 | 0.16 |
| Emotional Valence | -0.48 | 0.33 | -0.16 | 0.25 | 0.01 | -0.09 |

Supplementary Table 12.

*Content Domains as a Moderator of the Effect of Thinking Condition on Delay Discounting (ln(k))*

| Variable in interaction | β | Standard Error | CI - lower | CI - upper | p-value |
| --- | --- | --- | --- | --- | --- |
| CD1 | -0.11 | 0.13 | -0.36 | 0.13 | 0.367 |
| CD2 | 0.28 | 0.17 | -0.06 | 0.61 | 0.11 |
| CD3 | -0.28 | 0.18 | -0.64 | 0.08 | 0.134 |
| CD4 | 0.17 | 0.18 | -0.17 | 0.52 | 0.326 |
| CD5 | 0.1 | 0.21 | -0.3 | 0.51 | 0.62 |
| CD6 | -0.35 | 0.23 | -0.8 | 0.1 | 0.132 |
